# A new genus and species of Nannopodidae (Crustacea, Copepoda, Harpacticoida) from the Yellow Sea of South Korea

**DOI:** 10.3897/zookeys.984.52252

**Published:** 2020-11-04

**Authors:** Jong Guk Kim, Jimin Lee

**Affiliations:** 1 Marine Ecosystem Research Center, Korea Institute of Ocean Science & Technology, Busan 49111, South Korea Korea Institute of Ocean Science & Technology Busan South Korea

**Keywords:** benthic copepod, *Concilicoxa hispida* gen. et sp. nov., East Asia, meiofauna, taxonomy

## Abstract

A new monospecific genus of the family Nannopodidae Brady, 1880 is proposed, based on specimens of both sexes of *Concilicoxa hispida***gen. et sp. nov.** collected from subtidal sandy sediments in the Yellow Sea of South Korea. The presence of a coxal outer projection on the first to fourth legs and reduction of both rami of the second to fourth legs in this new genus show a clear relationship with a clade, which is characterised by the modified thoracopods for burrowing ability, comprising *Huntemannia* Poppe, 1884, *Rosacletodes* Wells, 1985, *Laophontisochra* George, 2002, *Acuticoxa* Huys & Kihara, 2010 and *Talpacoxa* Corgosinho, 2012 in Nannopodidae. Within this clade, *C. hispida***gen. et sp. nov.** is most closely related to *L. maryamae* George, 2002 in having the prehensile endopod in the first leg, broad intercoxal sclerite on the second to fourth legs and the female fifth leg being composed of separate exopod and baseoendopod, but is distinguished by the absence of mandibular exopod, two-segmented mandibular endopod, presence of four setae on the distal exopodal segment of the first leg, and fusion of the intercoxal sclerite to the coxae in the third and fourth legs. These four features are considered as autapomorphies of the new genus. The possible relationship amongst members of the nannopodid clade is further discussed. Additionally, some comments on the taxonomic position of *L. terueae* Björnberg, 2014 are given, resulting in the transfer of the species to *Acuticoxa* as *A. terueae***comb. nov.**

## Introduction

Por (1986) proposed the family Huntemanniidae Por, 1986 to accommodate six cletodid genera, *Nannopus* Brady, 1880, *Huntemannia* Poppe, 1884, *Pontopolites* T. Scott, 1894, *Metahuntemannia* Smirnov, 1946, *Beckeria* Por, 1986 (= a junior subjective synonym of *Metahuntemannia*) and *Pseudocletodes* T. Scott & A. Scott, 1893–the latter three genera with some reserves. Since its proposal, two genera, *Rosacletodes* Wells, 1985 [= *Echinocletodes* Pallares, 1982 *nec*Lang (1936)] and *Dahmsopottekina* Özdikmen, 2009 [= *Talpina* Dahms & Pottek, 1992 *nec*Hagenow (1840)], have been added to the family (Dahms and Pottek 1992; Huys et al. 1996; Wells 2007; Huys 2009). The family-group name Huntemanniidae was initially universally accepted (Huys et al. 1996; Boxshall and Halsey 2004; Wells 2007; Huys 2009); however, Huys (2009) recognised that Brady (1880) had assigned the new genus *Nannopus* to the new subfamily Nannopinae within Harpacticidae and that this genus could be considered the type genus of the latter subfamily. Therefore, Huys (2009) formally synonymised the family Huntemanniidae with Nannopodidae (adjusted name), considering the huntemanniid genera proposed by Por (1986) as valid members of the latter family. Subsequent studies questioned the monophyly of Nannopodidae and excluded three genera: Kihara and Huys (2009) transferred *Pseudocletodes* to the family Normanellidae Lang, 1944 and Huys and Kihara (2010)re-assigned both *Metahuntemannia* and *Dahmsopottekina* to the subfamily Hemimesochrinae Por, 1986.

Huys and Kihara (2010) also noted that *Laophontisochra* George, 2002, *Acuticoxa* Huys & Kihara, 2010, *Huntemannia* and *Rosacletodes* share the coxal modifications of the first leg and proposed the inclusion of two former genera into the family Nannopodidae. Subsequently, the nannopodid genus *Talpacoxa* Corgosinho, 2012 was established for an intriguing species, *T. brandini* Corgosinho, 2012, which is characterised by hypertrophied coxae of the first leg. According to Corgosinho (2012), these five nannopodid genera constitute a clade supported by the presence of an outer coxal projection on the first leg and reduction of both rami on the second to fourth legs as a morphological adaptation for a burrowing lifestyle.

Recently, Kim et al. (2017) proposed the revival of the genus *Ilyophilus* Lilljeborg, 1902, which had been considered a junior synonym of *Nannopus*, based on a morphological difference in the segmentation of endopod in the third leg, i.e. *Nannopus* could accommodate only two species, *N. palustrissensu stricto*Brady (1880) and *N. parvipilis* Kim, Choi & Yoon, 2017, having a one-segmented endopod and the other *Nannopus* species with a two-segmented endopod should be transferred into *Ilyophilus*. However, Vakati et al. (2019: 376) questioned the validity of re-instating *Ilyophilus*, based on morphological and molecular affinities between both genera. More recently, Lee (2020) proposed the new genus *Doolia* Lee, 2020 as a valid genus of the family Nannopodidae from off Jeju Island of South Korea. To date, the family Nannopodidae is composed of 30 valid species distributed in eight genera, *Nannopus*, *Huntemannia*, *Pontopolites*, *Rosacletodes*, *Laophontisochra*, *Acuticoxa*, *Talpacoxa* and *Doolia* (Kim et al. 2017; Vakati and Lee 2017; Karanovic and Cho 2018; Lee 2020).

In the present study, a new genus, attributed here to the family Nannopodidae, is proposed to accommodate a new harpacticoid collected from subtidal sandy sediments around the Socheongcho Ocean Research Station (SORS), which is a platform-type observation tower in the Yellow Sea of South Korea. SORS plays an important role in monitoring ocean and meteorological changes related to global climate change. Herein, we describe this new taxon and clarify its taxonomic relationship within Nannopodidae. Additionally, we also discuss the taxonomic position of *L. terueae* Björnberg, 2014.

## Materials and methods

**Figure 1.**
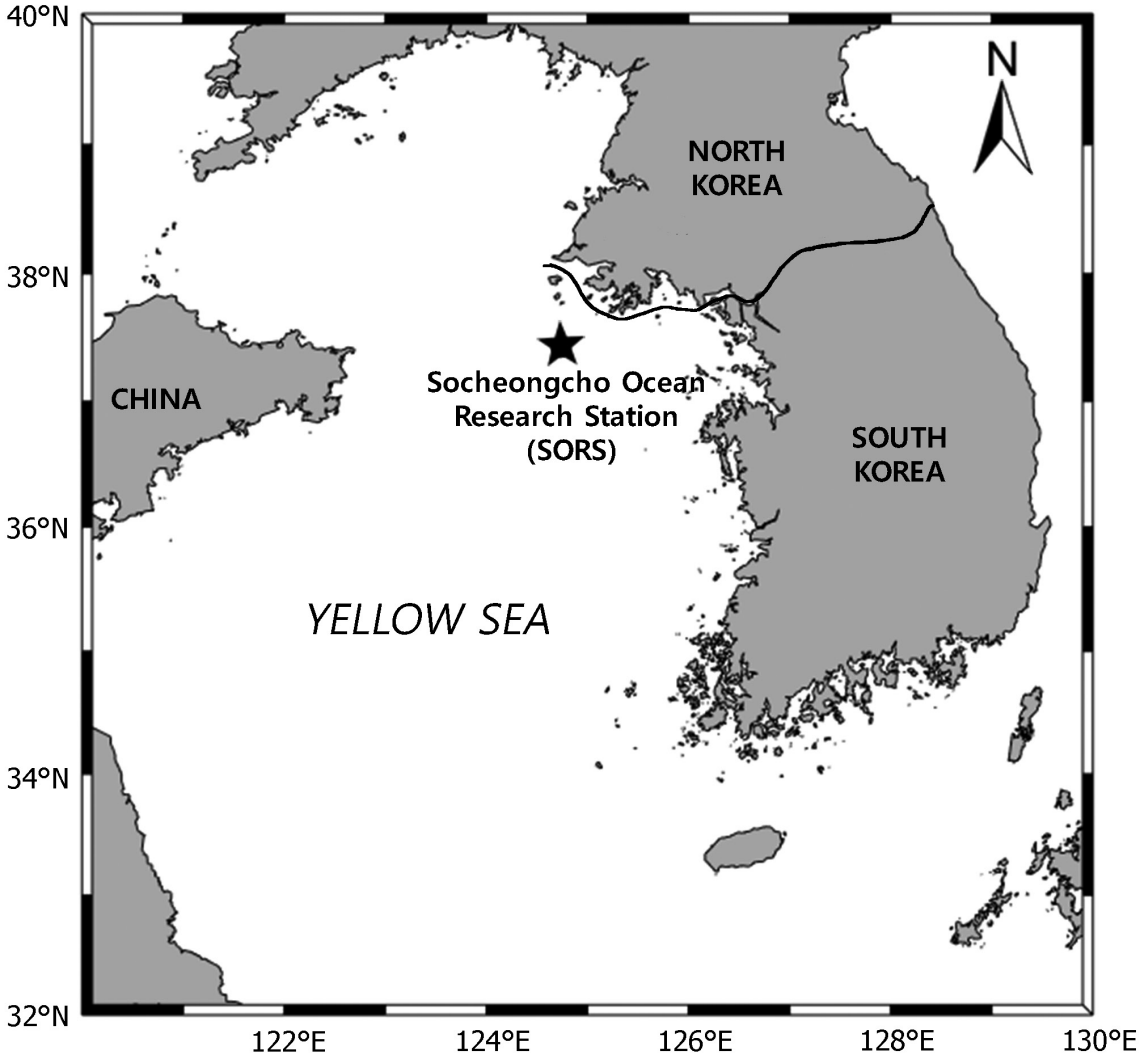
Map of the sampling location.

Sampling for meiofauna was carried out from off SORS in the Yellow Sea of South Korea (Fig. 1). Sediment sample was taken at a depth of 68 m using a Smith-McIntyre Grab sediment sampler (0.1 m^2^). To anaesthetise meiofaunal organisms, the sample was immediately bottled with a 7.5% MgCl_2_ solution for 1 h and fixed with a 10% formalin solution. In the laboratory, this sample was rinsed and sieved with tap water using a 50 μm sieve. Harpacticoid copepods were sorted out from sediments under a stereomicroscope (M165 C; Leica, Germany) and stored in 95% ethanol. Specimens of a new taxon were cleared in lactic acid. Whole specimens were mounted separately on temporary slides for habitus drawing and measurement of the total body length and the latter was measured from the anterior tip of the rostrum to the posterior end of the caudal rami in lateral view. Specimens were dissected on a reverse slide (Humes and Gooding 1964) using tungsten needles and the dissected parts were examined. All drawings were made with a microscope (DM2500; Leica, Germany) equipped with differential interference contrast (DIC) and a drawing tube. Drawings of the habitus and appendages were prepared at a magnification of 400× and 1000×, respectively. After morphological examination, each dissected part was mounted in lactophenol or fluoromount-G (SouthernBiotech, USA) mounting medium on an H-S slide (Shirayama et al. 1993) and sealed with Hoyer’s solution. Scale bars in figures are given in μm.

We adopted the descriptive terminology of Huys and Boxshall (1991). The following abbreviations were used in the text and figures:

**ae** aesthetasc;

**P1–P6** first to sixth thoracopod;

**exp(enp)-1(-2, -3)** to denote the proximal (middle, distal) segment of exopod (endopod).

Prior to scanning electron micrography (SEM), specimens were pre-fixed with 2.5% glutaraldehyde for 4 h, post-fixed with 2% osmium tetroxide for 2 h and then stored in 0.1 M phosphate buffer (pH 7.4) overnight. At each step, the samples were washed with phosphate buffer solution three times for 10 min each. The materials were dehydrated through a graded series of ethanol dilutions (50%, 60%, 70%, 80%, 90%, 100%) for 30 min each, dried in a freeze dryer (Hitachi ES-2030; Japan), coated with gold in an evaporator (Hitachi E-1045; Japan) and then examined via SEM (Hitachi S-4300; Japan).

Type materials were deposited in the Marine Interstitial fauna Resources Bank (MInRB) of the Korea Institute of Ocean Science and Technology (KIOST), Busan, South Korea.

## Systematics

### Family Nannopodidae Brady, 1880

#### Genus Concilicoxa gen. nov.

Taxon classificationAnimaliaHarpacticoidaNannopodidae

98A1F3ED-994A-5804-A61D-6FCCB2ABC0E8

http://zoobank.org/76CAD23D-83AE-4ADD-BA77-D21CB3E66CE5

##### Diagnosis.

Nannopodidae. Body subcylindrical, slightly depressed dorsoventrally, without distinct constriction between prosome and urosome; hyaline frills of somites weak, ornamented with long setules. Rostrum well-developed, triangular, with 1 pair of sensilla. Genital slit ♀ reverse U-shaped, covered by 1 pair of large opercula. Caudal rami elongate, oval in ♀, rectangular in ♂, with 7 setae; principal seta V as long as caudal rami in ♀, slightly shorter than urosome in ♂. Antennule 4-segmented, with elongate first segment in ♀; chirocerate and 6-segmented in ♂. Antennary allobasis without abexopodal seta; exopod represented by single seta. Mandibular palp uniramous, with 1 seta on basis; exopod absent and endopod 2-segmented. Maxillular praecoxal arthrite with 1 surface seta; exopod 1-segmented and endopod absorbed into basis. Maxillary syncoxa with 2 endites; endopod absorbed into basis. Maxilliped with elongate basis; endopod drawn out into long geniculate claw. P1 coxa with 1 coarsely serrate outer projection; inner element on basis displaced onto anterior surface; exopod 2-segmented, with 4 setae on exp-2; endopod arising from well-developed inner pedestal of basis, prehensile, 2-segmented; enp-1 elongate, unarmed; enp-2 with 1 stout claw and 1 long seta. P2–P4 with 1 coarsely serrate outer projection on coxa; intercoxal sclerite hugely broad, separated in P2 and laterally fused to coxae in P3 and P4; outer setophore on basis articulated in P3 and P4; exopod 1-segmented; proximal outer spine on exopod with serrate outer margin, with inner and outer longitudinal rows of setules; endopod absent in P2, represented by 1 small distinct protuberance in P3, 1-segmented in P4; male P3 endopod 1-segmented and armed with 1 stout spine. Setal armature formulae of P1–P4 as follows:

**Table:** 

	Exopod	Endopod
P1	0.022	0.011
P2	022	absent
P3	112	000 [010 in ♂]
P4	112	010

P5 baseoendopod broad; endopodal lobe weakly developed, with 1 seta; exopod 1-segmented, with 4 setae.

P6 represented by 2 setae in ♀; slightly asymmetrical and represented by 3 setae in ♂.

##### Type species.

*Concilicoxa hispida* gen. et sp. nov., by monotypy.

##### Etymology.

The generic epithet is a combination of the Latin verb *concílĭo* meaning ‘unite separate parts into a whole’ and the Latin noun *coxa*, meaning ‘hip’ and alludes to the fusion of the coxae and the intercoxal sclerite in P3 and P4. It is a noun in the feminine singular.

#### Concilicoxa hispida

Taxon classificationAnimaliaHarpacticoidaNannopodidae

gen. et sp. nov.

BB01C12B-7EFC-5670-95AF-26E857BF3C8F

http:///zoobank.org/06ABD568-8291-43BC-8AC7-0A6FE8F9BECC

**Figure 2.**
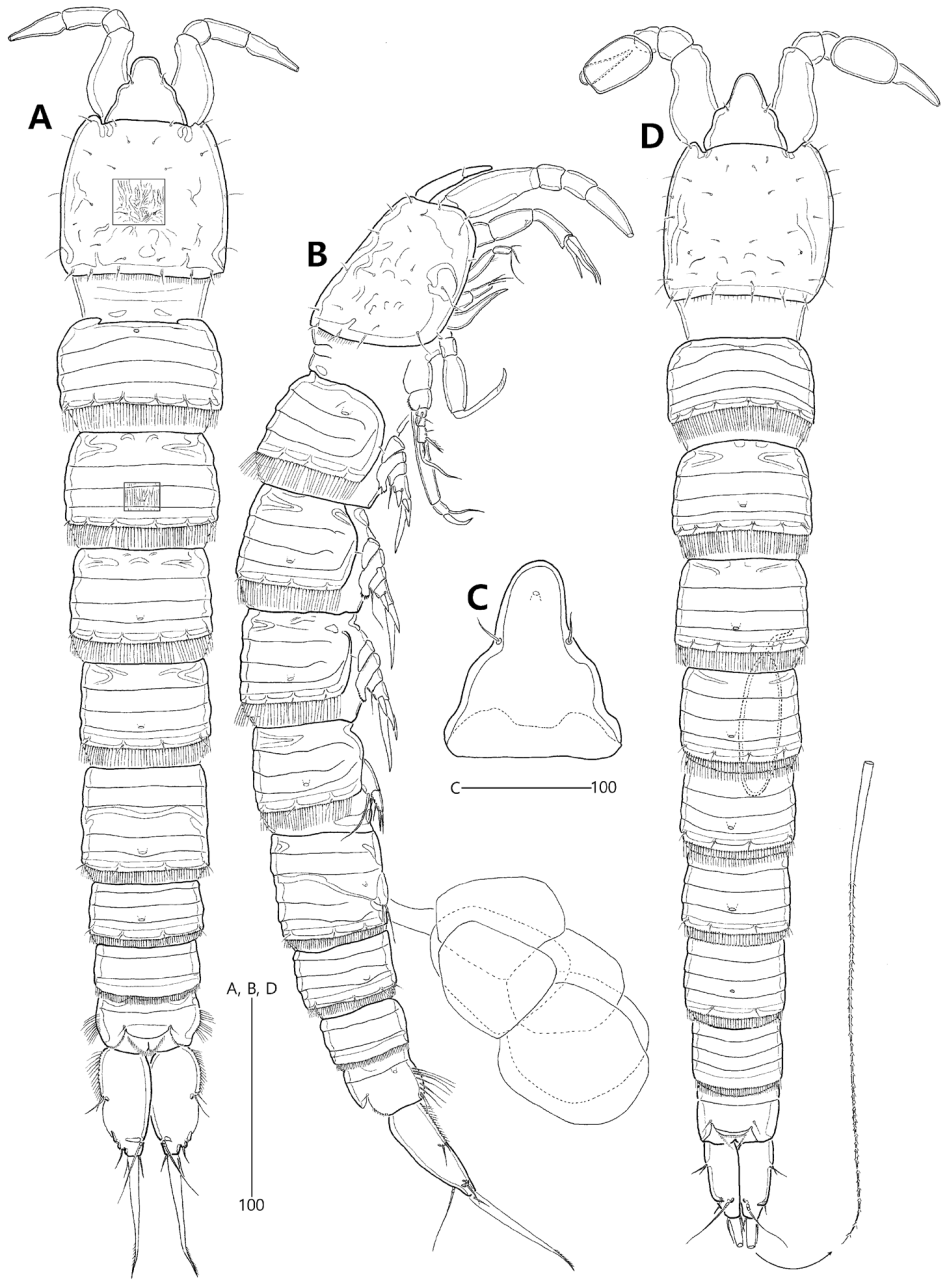
*Concilicoxa hispida* gen. et sp. nov., female holotype (**A–C**) **A** habitus, dorsal **B** habitus, lateral **C** rostrum, dorsal. Male allotype (**D**) **D** habitus, dorsal.

**Figure 3.**
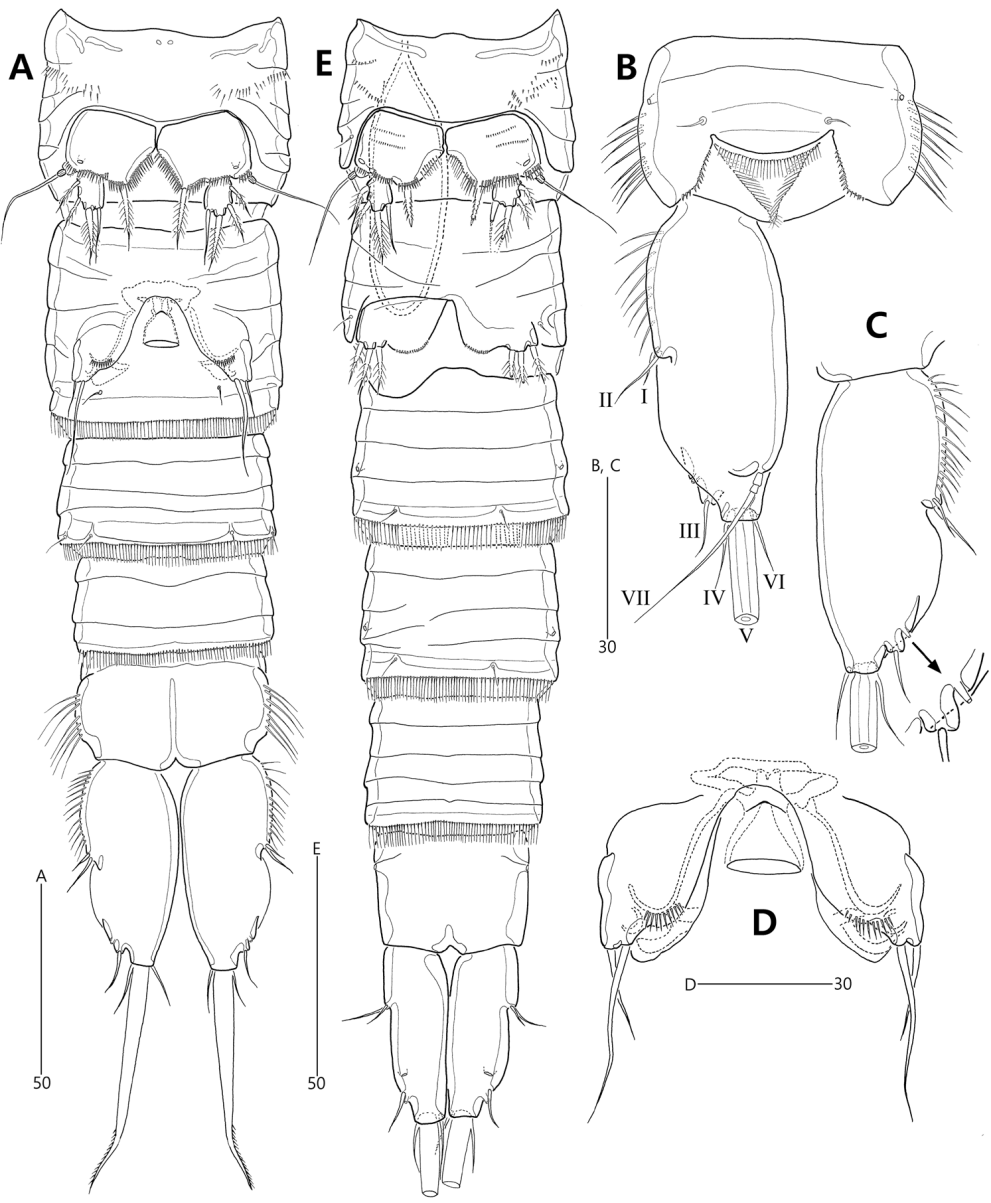
*Concilicoxa hispida* gen. et sp. nov., female holotype (**A–D**) **A** urosome, ventral **B** anal somite and caudal ramus, dorsal **C** caudal ramus, ventral **D** genital field, ventral. Male allotype (**E**) **E** urosome, ventral.

**Figure 4.**
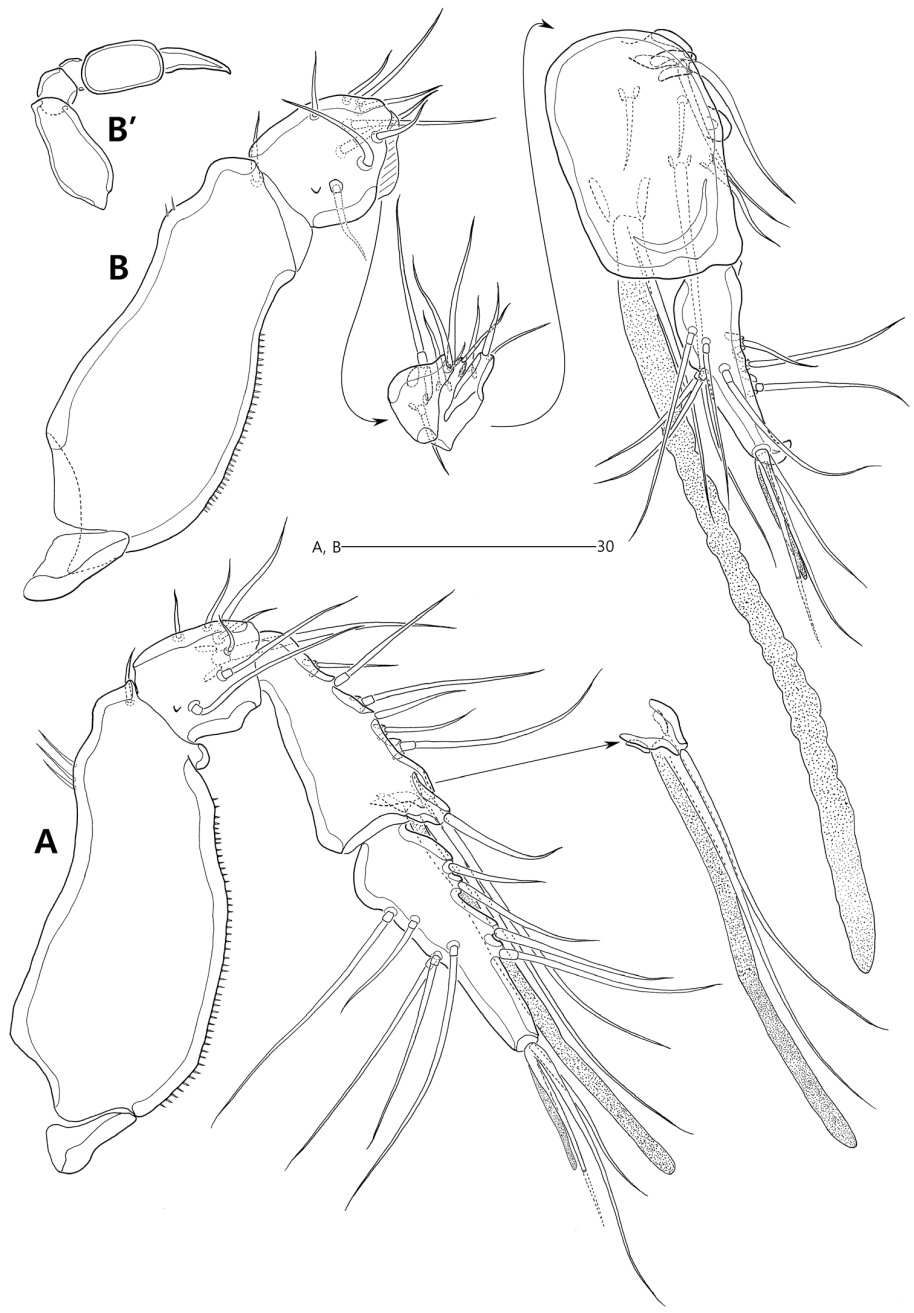
*Concilicoxa hispida* gen. et sp. nov., female holotype (**A**) **A** antennule. Male allotype (**B**) **B** antennule.

**Figure 5.**
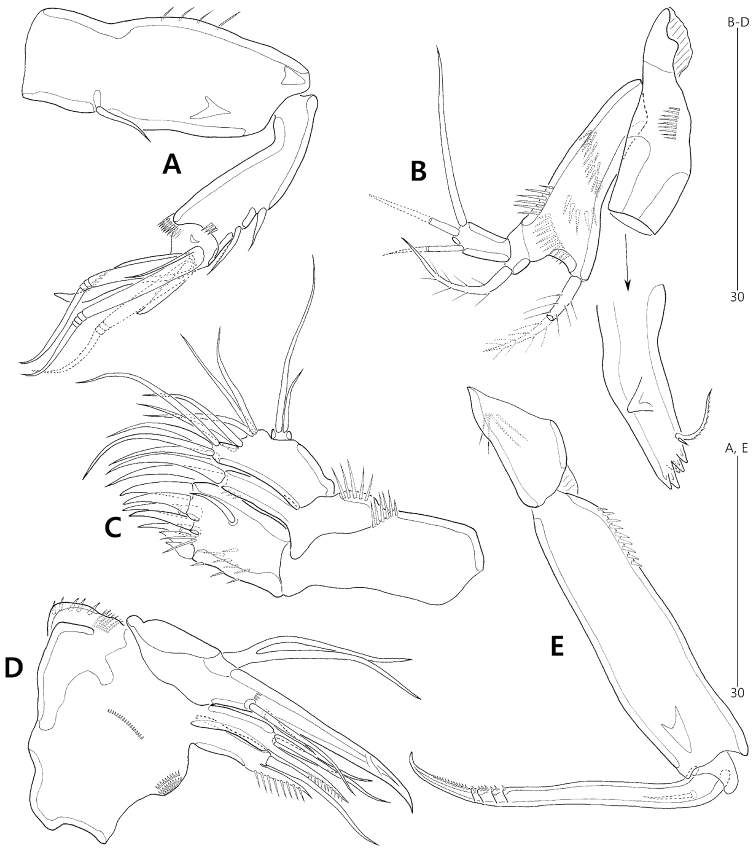
*Concilicoxa hispida* gen. et sp. nov., female holotype. **A** antenna (lacking coxa) **B** mandible **C** maxillule **D** maxilla **E** maxilliped.

**Figure 6.**
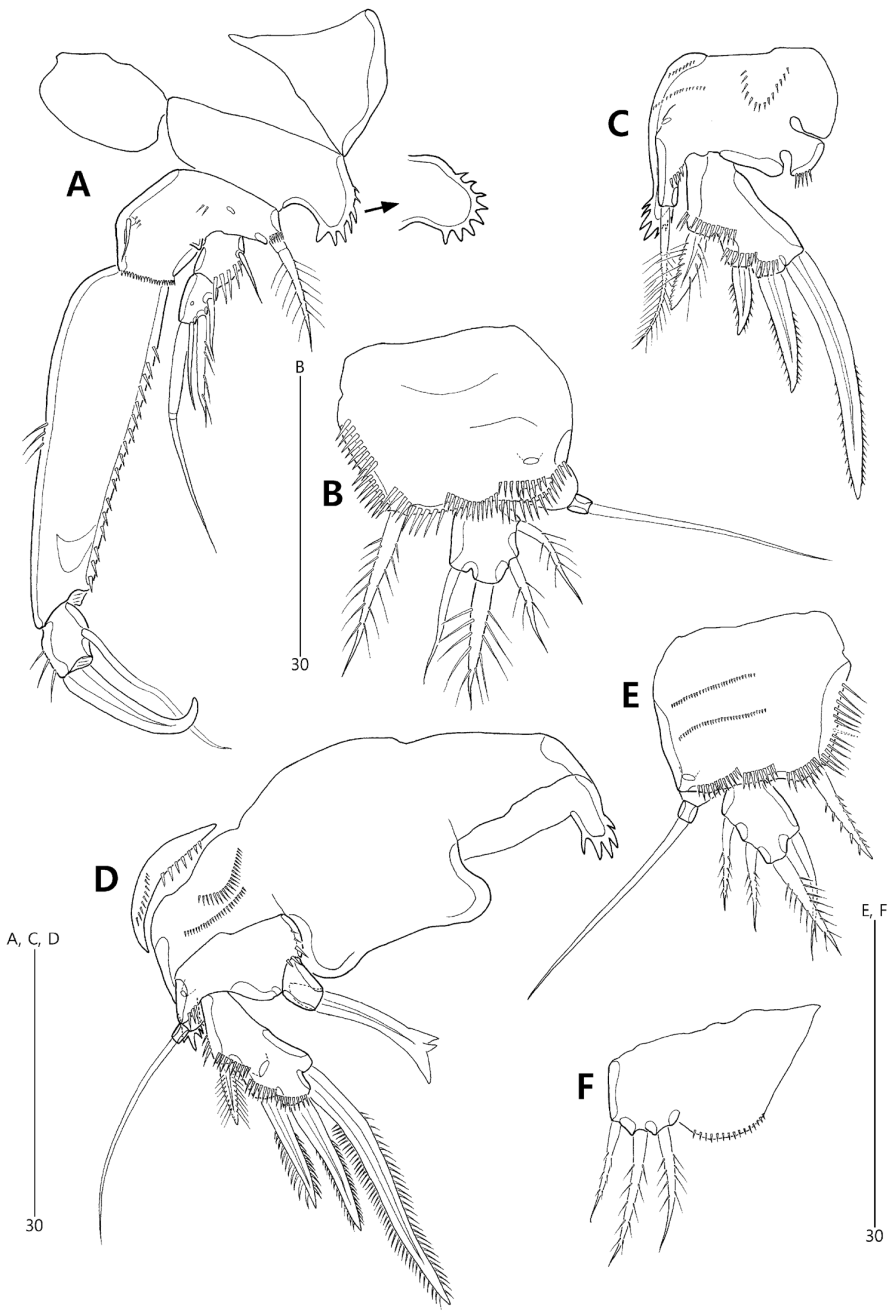
*Concilicoxa hispida* gen. et sp. nov., female holotype (**A–B**) **A**P1, anterior **B**P5, anterior. Female paratype (**C**) **C** abnormality of P2, anterior. Male allotype (**D–F**) **D**P3, anterior **E**P5, anterior **F**P6, anterior.

**Figure 7.**
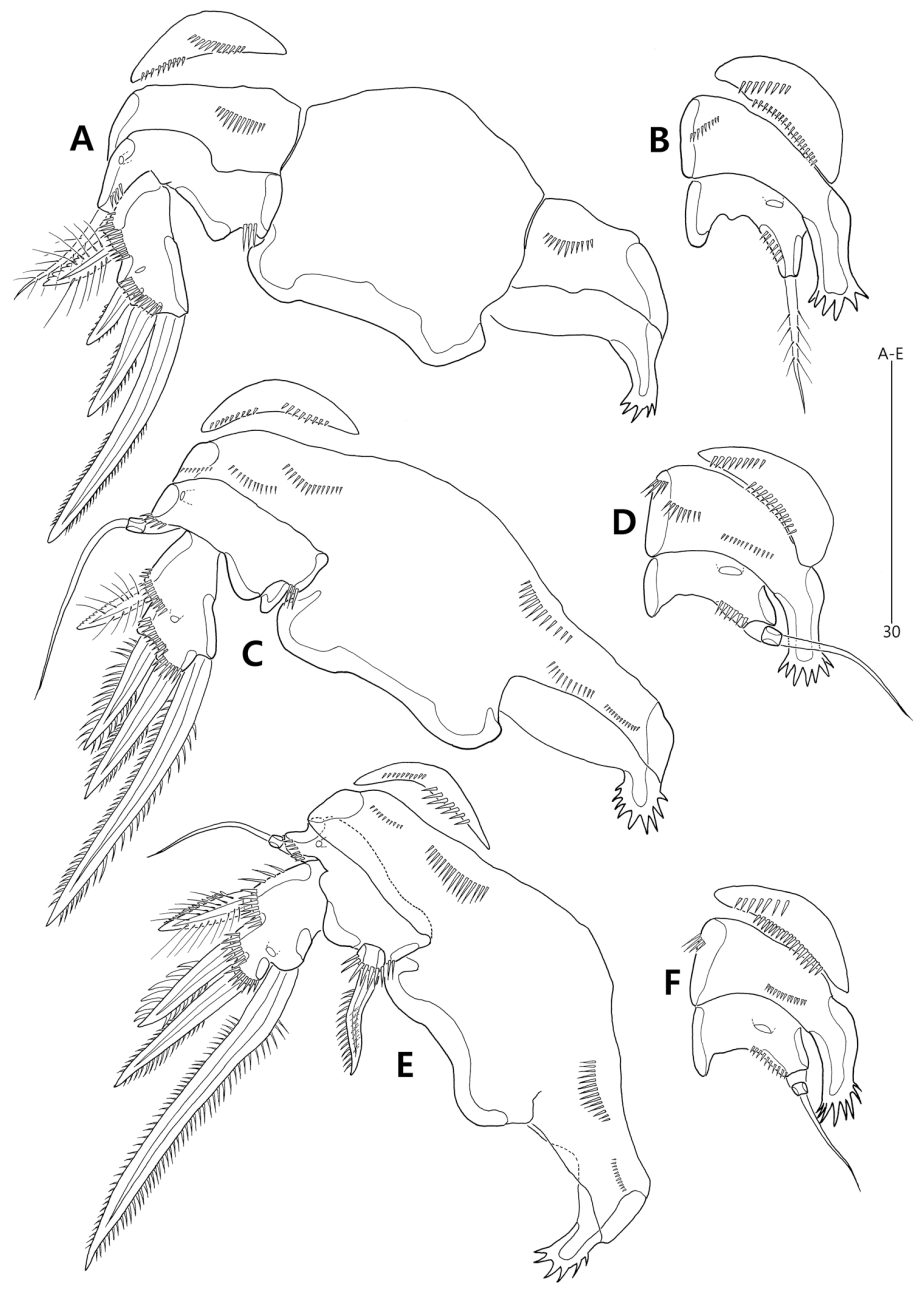
*Concilicoxa hispida* gen. et sp. nov., female holotype **A**P2, anterior **B** protopod of P2, lateral **C**P3, anterior **D** protopod of P3, lateral **E**P4, anterior **F** protopod of P4, lateral.

**Figure 8.**
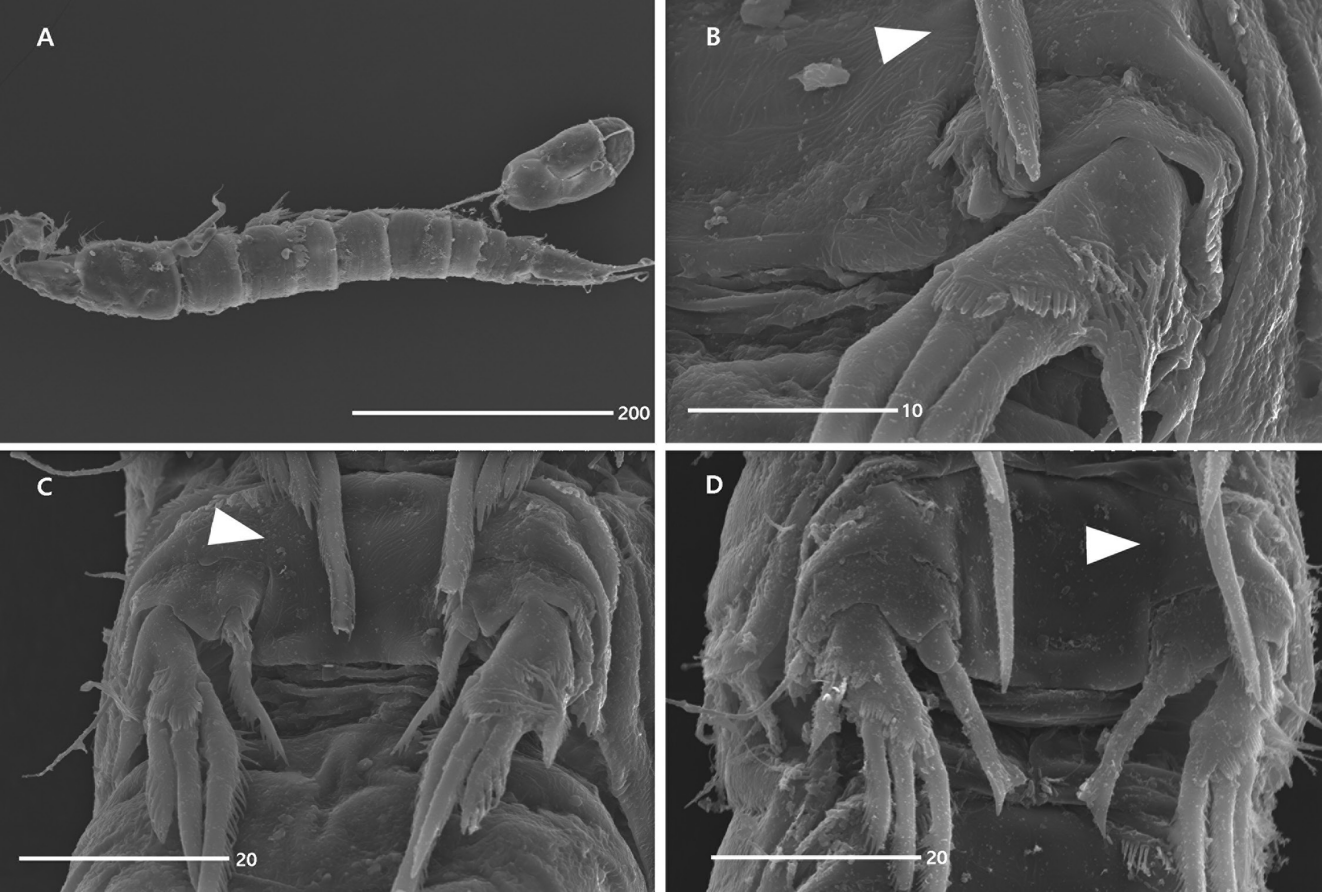
*Concilicoxa hispida* gen. et sp. nov., scanning electron micrograph, female (**A–C**) **A** habitus, lateral **B**P3, anterior **C**P4, anterior. Male (**D**) **D**P3, anterior. Arrowheads indicate fusion of coxa and intercoxal sclerite.

Figs 2 , 3 , 4 , 5 , 6 , 7 , 8

##### Type locality.

Off the Socheongcho Ocean Research Station (SORS) (37°25'57.16"N, 124°44'56.4"E) in the Yellow Sea of South Korea, sandy sediments, 68 m depth.

##### Material examined.

***Holotype***: SOUTH KOREA•♀ dissected and mounted on 11 slides; the Yellow Sea, off SORS; 37°25'57.16"N, 124°44'56.4"E; 68 m depth; 23 Mar 2018; Kim, J.G. leg.; sandy sediments; cat. MInRB-Hr59-S001.

***Allotype***: SOUTH KOREA•♂ dissected and mounted on 11 slides; same data as for holotype; cat. MInRB-Hr59-S002.

***Paratypes***: SOUTH KOREA•3♀♀2♂♂ dissected and mounted on 11 or 12 slides each; same data as for holotype; cat. MInRB-Hr59-S003–MInRB-Hr59-S007•3♀♀2♂♂ preserved together in 95% ethanol; same data as for holotype; cat. MInRB-Hr59-L001.

##### Other material for SEM.

SOUTH KOREA•2♀♀1♂ on a stub for SEM; same data as for holotype.

##### Description of holotype female

(MInRB-Hr59-S001). Total body length 617 μm (measurement based on holotype and six paratypes: range = 530–626 μm; mean = 588 μm; *n* = 7); maximum width 86 μm measured at the middle of cephalothorax. Body (Figs 2A, B, 8A) subcylindrical, slightly depressed, without distinct constriction between prosome and urosome; prosome slightly longer than urosome. Rostrum (Fig. 2C) well-developed, triangular, reaching distal fourth of first antennular segment, defined from cephalothorax basally, with 1 pair of sensilla laterally and 1 median anterior pore ventrally; lateral margins convex proximally. Cephalothorax nearly square in dorsal aspect, slightly wider than long; integument covered with paired sensilla, several round depressions and irregular wrinkles (visible at high magnification, 1,000×; see insert in Fig. 2A); posterior margin ornamented with short and fine setules; arthrodial membrane of first pedigerous somite visible dorsally and laterally. Tergites of somites with surface ornamentation composed of 1–3 transverse furrows, with 1 mid pore (absent in penultimate and anal somites) and 1 pair of lateral pores (absent in penultimate somite); posterior margins with several paired sensilla (absent in penultimate somite); hyaline frills weak, with 1 row of long setules posteriorly except for anal somite. Genital somite and first abdominal somite fused ventrally forming genital double-somite, but original segmentation indicated by internal chitinous rib dorsally and laterally; genital field (Fig. 3A, D) with 1 large copulatory pore on midventral depression posterior to genital slit; genital slit reverse U-shaped, covered by 1 pair of large opercula derived from P6 on both sides; P6 represented by 1 long and 1 small seta, with 1 row of spinules subdistally; single midventral egg sac carrying 6 large eggs, as long as 1/4 of total body length. Anal somite (Figs 2A, B, 3A, B) with 1 pair of dorsal sensilla near base of operculum, 1 row of long setules on both ventrolateral margins; operculum semicircular, with smooth distal margin; anal opening with lateral row of small posterior spinules on each side; anal opening with 3 fringes of fine setules (Fig. 3B).

Caudal rami (Figs 2A, B, 3A–C) elongate, oval, about 2.4 times as long as largest width, twice as long as anal somite; with a notch in mid-outer margin below caudal setae I and II; anterior half with a row of outer setules ventrolaterally; distal half with non-chitinous lateral margin; with 7 setae: seta I small naked, inserted in mid-length of outer margin ventrolaterally; seta II dorsal to and closely set to seta I, naked, longer than seta I; seta III naked, as long as seta II, arising from subdistal peduncle with 1 tube pore basally (Fig. 3C); seta IV small, naked, slightly longer than setae II and III, fused to principal seta V basally; principal seta V well-developed, slightly longer than caudal ramus, ornamented with outer spinules distally; seta VI naked, as long as seta IV, inserted in outer distal corner; dorsal seta VII naked, tri-articulate at base, arising subdistally close to inner margin.

Antennule (Fig. 4A) short, 4-segmented. First segment largest, elongate, as long as distal two segments combined, with 1 small naked seta subdistally; inner margin with short row of spinules subdistally; outer margin convex, with longitudinal row of minute spinules. Second segment smallest, with 3 bi-articulate and 5 naked setae; outer margin with 1 weak protuberance. Third segment about twice as long as second one, gradually widening distally; lateral margin with 3 bi-articulate and 3 naked setae; inner distal corner with 3 peduncles, of which two with 1 apical seta each, and one bearing 1 apical seta fused to basally to 1 ae. Distal segment as long as preceding one, tapering distally; lateral margins with 6 bi-articulate and 3 naked setae; distal margin with 1 naked seta and 1 acrothek composed of 1 ae and 2 bare setae. Setal armature as follows: 1-[1], 2-[8], 3-[8+ (1 + ae)], 4-[10 + acrothek].

Antenna (Fig. 5A) with small, unornamented coxa (not shown). Allobasis elongate, 2.8 times as long as wide, exopod represented by 1 naked seta issuing at proximal third; abexopodal seta absent. Free endopodal segment with 1 short row of spinules subdistally and 1 surface frill distally; lateral armature composed of 2 weakly-serrate setae; distal armature comprising 1 small and 1 stout spine, 3 geniculate setae, innermost one of which fused basally to 1 small naked seta.

Mandibular coxa (Fig. 5B) slender, with 1 bulge and 1 row of spinules proximally; gnathobase well-developed, with 1 bicuspid and 3 unicuspid teeth, 1 small spinule and 1 unipinnate seta. Palp well-developed, uniramous; basis elongate, covered with rows of spinules, with 1 plumose seta distally; endopod 2-segmented, with 1 long plumose seta on proximal segment and 1 subapical and 2 apical setae on distal segment.

Maxillule (Fig. 5C). Praecoxa with 1 row of outer spinules; arthrite with 1 naked seta on anterior surface and 7 spines on distal margin and ornamented with few long spinules on distal margin, 1 row of small spinules on dorsal margin and several spinules on posterior surface. Coxa armed with 1 row of outer spinules; endite elongate, with 2 elements distally and 1 row of small spinules laterally. Basis broad, with 2 endites: distal endite with 1 subapical and 3 apical setae; proximal endite incorporated into basis, represented by 2 long naked setae. Endopod incorporated into basis, represented by 3 long naked setae. Exopod 1-segmented, small, with 1 short and 1 long naked seta.

Maxilla (Fig. 5D). Syncoxa armed with 1 row of stout spinules and 1 row of setules along outer margin, 1 row of minute spinules on surface and 1 patch of spinules near inner margin; with 2 coxal endites: proximal endite with 1 long naked seta and 1 short unipinnate seta (fused to endite basally); distal endite with 2 long naked setae and 1 unipinnate seta (fused to endite basally). Allobasis drawn out into strong claw with 2 accompanying naked setae and few spinules. Endopod incorporated into basis, represented by 2 long naked setae fused basally.

Maxilliped (Fig. 5E) enlarged. Syncoxa elongate, ornamented with 1 group of spinules proximally. Basis elongate, about 3.4 times as long as maximum width, with 1 row of outer spinules proximally. Endopod drawn out into long and geniculate claw bearing 1 small accessory seta proximally.

P1 (Fig. 6A). Praecoxa large, triangular, unornamented. Intercoxal sclerite broad, unornamented. Coxa wide, with outer margin forming 1 large and coarsely-serrated projection. Basis with 1 anterior pore and few spinules proximally; inner pedestal well-developed, with serrate distal margin; outer seta plumose, bi-articulated basally, arising from setophore ornamented with 1 row of small spinules at its base; inner seta naked, arising anteriorly, with 1 group of small spinules at its base. Exopod 2-segmented, short, about 0.3 times as long as enp-1; exp-1 with 1 naked outer seta and 1 row of stout outer spinules; exp-2 with 1 small naked seta and 1 stout unispinulose seta on outer margin and 1 short naked and 1 long geniculate seta on distal margin; anterior surface with 1 small pore. Endopod prehensile, 2-segmented; enp-1 elongate, 3.7 times as long as largest width, ornamented with 1 row of small spinules along outer margin and few long inner spinules; enp-2 short, slightly longer than wide, ornamented with few inner spinules and armed with 1 stout, recurved distal claw and 1 long flexible outer seta, of which distal half with very thin cuticular inner lines.

P2–P4 (Figs 7A–F, 8B, C). Protopods composed of praecoxa, coxa and basis. Praecoxae small, ornamented with 2 rows of spinules. Intercoxal sclerites large, broad, separate in P2, fused to coxae laterally in P3 and P4 (see arrowheads in Fig. 8B, C). Coxae wide, with 1, 3 and 2 groups of spinules on anterior surface in P2–P4, respectively; outer margin drawn out into an elongate and coarsely-serrated projection. Bases wide, with 1 pore on anterior surface; outer setophore elongate, ornamented with 1 row of spinules basally, non-articulated in P2 with 1 plumose seta, bi-articulated in P3 and P4, with 1 naked seta; inner distal corner (near base of inner ramus) with 1 group of spinules. Exopod 1-segmented, ornamented with rows of spinules along outer and distal margins; with 4 stout outer spines, of which proximal one uniserrate and ornamented with 2 rows of setules, others pinnate; distal outer spines of P4 strongly pinnate. Endopod absent in P2, represented by 1 small unarmed protuberance in P3 and 1-segmented, ornamented with distal spinules and armed with 1 stout pinnate distal spine in P4.

P5 (Fig. 6B). Baseoendopod broad, with 1 anterior pore, ornamented with rows of spinules along distal and inner margins; endopodal lobe weak, with 1 plumose distal seta; outer setophore articulate, with 1 long naked seta. Exopod small, with 3 pinnate setae and 1 naked seta.

***Male*** (allotype MInRB-Hr59-S002). Total body length slightly shorter than in female, 525 μm (measurement based on allotype and 4 paratypes: range = 485–556 μm; mean = 512 μm, n = 5); body (Fig. 2D) slightly more slender than in female, maximum width 74 μm measured at the middle of cephalothorax; urosome 6-segmented, comprising P5-bearing somite, genital somite, 3 abdominal somites and anal somite; penultimate somite slightly shorter than its width, without lateral ornamentation. Caudal rami (Figs 2D, 3E) parallel, rectangular, more slender than in female; inner margin straight, outer margin slightly convex; outer margins unornamented, with clear cuticular inner line; additional large pore present on ventral surface; seta III issuing from subdistal lateral margin ventrally; set of setae I and II issuing from proximal third of outer margin; seta V slightly shorter than urosome (Fig. 2D).

Antennule (Fig. 4B) chirocerate, 5-segmented. First segment elongate, with 1 short naked seta subdistally; inner margin with few small spinules; outer margin convex, with 1 row of minute spinules. Second segment slightly longer than wide, with 2 bi-articulate and 7 naked setae and 1 minute protuberance. Third segment partially separated into two parts; proximal one with 2 bi-articulate and 6 naked setae; distal part with 2 setae. Fourth segment swollen, with 1 medial protuberance, 4 naked surface setae and 3 well-developed posterior penduncles: one proximal and one medial peduncle with 1 long naked apical seta each; subdistal peduncle with 1 long naked seta fused to 1 long ae basally. Distal segment elongate, slightly recurved distally, hook-shaped, with 2 naked and 6 bi-articulate setae laterally, 1 long naked seta distally and 1 acrothek composed of 1 ae and 2 naked setae fused basally. Setal armature as follows: 1-[1], 2-[9], 3-[10], 4-[6 + (1 + ae)], 5-[10 + acrothek].

P3 (Fig. 6D) as in female, except for 1-segmented endopod with 1 stout distal spine bearing 2 pointed lateral processes.

P5 (Figs 3E, 6E) as in female except for anterior ornamentation with 2 rows of minute spinules.

P6 (Figs 3E, 6F) asymmetrical (one side completely fused to genital somite basally, other side articulated at base), each represented by a plate bearing 3 plumose setae and 1 row of minute spinules distally on inner extension.

Spermatophore as long as 4/5 length of P5-bearing and genital somites combined (Figs 2D, 3E).

##### Etymology.

The species epithet “*hispida*” is derived from the Latin adjective *híspĭdus*, which means ‘hairy’ and refers to the setulose lateral ornamentation of the anal somite and caudal rami in the female. It is a noun in the feminine singular.

##### Variability and abnormality.

The investigated individuals of *Concilicoxa hispida* gen. et sp. nov. show intraspecific differences in appendage ornamentation. Dense spinular ornamentation was observed on the mandibular basis in one female paratype (MInRB-Hr-59-S003). This paratype also displays fusion of the coxa and basis of the P2 symmetrically (Fig. 6C).

##### Remarks.

George (2002) established the genus *Laophontisochra* to accommodate *L. maryamae* from the Patagonian continental slope (Chile) and *Laophontisochra* sp. from the Magellan Straits (Chile). He allocated this genus into the family Cristacoxidae Huys, 1990, based on the presence of an outward growth on the coxa of P1, an enlarged maxilliped and atrophy of the antennary exopod and abexopodal seta despite the discrepancies with the following characters of the family Cristacoxidae, which were defined by Huys (1990): the first antennular segment with an outer spinous process, the absence of the exopod and an abexopodal seta in the antenna, the presence of modified seta on the middle endite of maxillary syncoxa and the single plate P5 with the same setae/spines in both sexes, which is considered as a neotenous origin. George (2002) suggested that the Cristacoxidae could be divided into two lineages: a plesiomorphic group comprising only *Laophontisochra* and a derived group composed of *Noodtorthopsyllus* Lang, 1965, *Cubanocleta* Petkovski, 1977 and *Cristacoxa* Huys, 1990 [the latter was considered as a junior synonym of *Noodtorthopsyllus* by Huys and Kihara (2010)]. However, Huys and Kihara (2010) transferred the genus *Laophontisochra* to the family Nannopodidae, based on a re-evaluation of the three fundamental morphological differences between the two groups suggested by George, with their newly-erected genus *Acuticoxa* within the family Nannopodidae for Laophontisochra sp. sensu George, 2002 (= *A. biarticulata* Huys & Kihara, 2010) and *A. ubatubaensis* Huys & Kihara, 2010 from the Brazilian coast. They assumed that both genera differ from the Cristacoxidae with the following evidence: (1) P1 coxa with a pair of serrated cristae (outer projections) in *Noodtorthopsyllus* and *Cubanocleta* vs. a single non-serrate, lobate or spinulose outgrowth in *Laophontisochra* and *Acuticoxa*; (2) maxillipedal endopod represented by a geniculated claw in *Laophontisochra* and *Acuticoxa* vs. non-geniculated in *Noodtorthopsyllus* and *Cubanocleta*; (3) antennary exopod consistently absent in *Noodtorthopsyllus* and *Cubanocleta* vs. atrophied in *Laophontisochra* and *Acuticoxa* (see Huys and Kihara 2010: 34). In addition, Huys and Kihara (2010) suggested that *Laophontisochra* and *Acuticoxa* are more closely related to both *Huntemannia* and *Rosacletodes* than to the cristacoxid genera, in that they share the presence of a coxal projection on the P1–P4 (vs. the plesiomorphic state of this character expressed in *Laophontisochra*, which lacks the coxal processes in the P2–P4). Corgosinho (2012) created the genus *Talpacoxa*, which was first mentioned as “Genus X” by Huys and Kihara (2010) and revealed close relationships amongst the genera of the nannopodid clade–*Huntemannia*, *Rosacletodes*, *Laophontisochra*, *Acuticoxa* and *Talpacoxa*–supported by three synapomorphies that are likely morphological adaptations to a burrowing lifestyle: (1) P1 coxa with an outer projection; (2) the P2–P4 exopods one- or two-segmented; and (3) the P2–P4 endopods one-segmented or vestigial.

The new genus *Concilicoxa* gen. nov. is assigned to the Nannopodidae because, as a member of the nannopodid clade, it exhibits the burrowing adaptation of the thoracopods. *Concilicoxa* gen. nov. appears to be closely related to both *Laophontisochra* and *Acuticoxa* in that they share four-segmented female antennules with elongate first segments, the prehensile P1 endopod, the presence of coxal outer projection on the P1, large and broad intercoxal sclerites on the P2–P4, the general shape of the female genital field (with a large copulatory pore and a well-developed operculum derived from P6) and elongate caudal rami. However, the novel genus is easily distinguishable from *Laophontisochra* by the distal armature of the antennary endopod with three geniculate and three non-geniculate elements (vs. four geniculate and two non-geniculate elements in *Laophontisochra*), the presence of coxal outer projections in the P2–P4 (vs. absent in *Laophontisochra*) and one-segmented exopods in the P2–P4 (vs. two-segmented in *Laophontisochra*). The new genus is also different from *Acuticoxa* in the absence of the P2 endopod (vs. one-segmented in *Acuticoxa*), a serrate coxal outer projection in the P1–P4 (vs. acute in *Acuticoxa*) and the female P5 exopod and baseoendopod separate (vs. fused into a single plate in *Acuticoxa*).

In contrast to a close resemblance with both genera in habitus and thoracopod morphology, *Concilicoxa* gen. nov. displays unambiguous autapomorphies that require the formation of a new genus: (1) the loss of the mandibular exopod, as observed in *Huntemannia*, is more derived than the exopod represented by a single seta; (2) the mandibular endopod is two-segmented, which seems to be secondarily divided, comparing to other related genera with only one-segmented endopod; (3) the P1exp-2 comprises a total of only four elements, but five or six setae in *Laophontisochra* and *Acuticoxa*, respectively (in the original description of *L. terueae*, this segment was described as having one lateral and three terminal setae, but was depicted as having three outer and three terminal elements; see Björnberg 2014: fig. 11A); and (4) the intercoxal sclerites of P3 and P4 are laterally fused with the coxae in *Concilicoxa* gen. nov. (Figs 6D, 7C, E, 8B–D), but this fusion has rarely been reported in harpacticoid copepods (i.e. *Orthopsyllus* sp. of the family Orthopsyllidae Huys, 1990; cf. Huys and Boxshall 1991). By contrast, the presence of the maxillular exopod, as observed in *Talpacoxa* demonstrates a more plesiomorphic state than the lack of endopod.

The males of *Concilicoxa* gen. nov. exhibit distinctive potential autapomorphies for the genus as follows: (1) the P3 endopod has a sexual dimorphic distal element that is a robust spine; (2) the shape of P5 is nearly similar to that of the female; and (3) the caudal rami show sexual dimorphisms in the length of caudal seta V, the issuing position of setae I and II and the number of tube pores. However, we could not compare these characters with other related genera, because males of *L. maryamae* and *A. ubatubaensis* remain unknown. The sexual dimorphism of thoracopods is one of the most robust characters used to assess the phylogenic relationships between genera and between families because it facilitates comparison of the positions of homologue elements (such as setae or apophyses) of rami in females and males (Huys 1990; Huys and Kihara 2010). In this nannopodid clade, the known males tend to exhibit differences in morphology of the P3 endopod: (1) the male of *Rosacletodes* has a two-segmented P3 endopod with an elongate inner apophysis on enp-2, instead of a single seta as in the female (Pallares 1982); (2) all known males of the species of *Huntemannia* have an additional armature element on the P3 endopod, with no differences in segmentation as in *Nannopus* and *Pontopolites* (Song et al. 2007; Karanovic and Cho 2018); (3) the male of *T. brandini* exhibits a distal small apophysis on the one-segmented P3 endopod; and (4) although the male of *L. maryamae* has yet to be discovered, there is no sexual dimorphism on the P3 in *L. terueae*, whose taxonomic position seems to be problematic (see below). The male P3 endopod of the new genus presented herein is one-segmented with a stout spine (Figs 6D, 8D), whereas the female P3 endopod is represented by an unarmed protrusion. Such diverse sexual dimorphism of the P3 endopod prevents deeper insight into the systematic position of this clade within the Nannopodidae. We hypothesise that the lack of original outer element on the female P3 endopod in *L. maryamae* and *C. hispida* gen. et sp. nov. leads to the absence of the sexual dimorphic apophysis in the male. In contrast, the presence of a small apophysis on the corresponding ramus in *T. brandini* seems to be derived from a rudimental apical seta in the female.

Harpacticoids generally display sexual dimorphism in the size, shape and setae of the male P5. However, no sexual dimorphism has been observed in the male P5 of Arenopontiidae Martínez Arbizu & Moura, 1994 (Martínez Arbizu and Moura 1994). Additionally, both sexes bear the same number of setae/spines on the P5 of some taxa, such as Metidae Boeck, 1873, Rotundiclipeidae Huys, 1988, Ectinosomatidae Sars, 1903 and Cristacoxidae Huys, 1990 (Huys 1988; Fiers 1992; Clément and Moore 1995; Huys and Kihara 2010). Except for Ectinosomatidae, the P5 of these families is remarkably reduced or represented by a single plate in both sexes. Although this sexual dimorphism is observed in other nannopodid genera, the structure of this leg in our new taxon is very similar in both sexes, except for micro-ornamentation, such as cuticular spinules and pores (Fig. 6B, E). In addition, the male of *Concilicoxa* gen. nov. expresses rare sexual dimorphisms in the shape of the caudal rami (oval in the female, but rectangular in the male), the length of caudal seta V (slightly longer than the caudal ramus in the female, but slightly shorter than the urosome in the male), the number of pores on the surface (one pore in the female vs. two pores in the male) and the lateral ornamentation (the presence of a row of long setules proximally in the female vs. absent in the male). These sexual dimorphisms could support the erection of a new genus *Concilicoxa* gen. nov.

## Discussion

### Taxonomic position of *Laophontisochra terueae* Björnberg, 2014

Björnberg (2014) described the second species of *Laophontisochra* (*L. terueae*) from the south-eastern coast of Brazil, but provided insufficient description and illustrations. She argued that *L. terueae* fits the generic diagnosis of the genus as amended by Huys and Kihara (2010), despite obvious differences in the presence of endopods in P2 and P3 and in the structure of the female P5. Huys and Kihara (2010) suggested that two species of *Acuticoxa*, *A. biarticulata* and *A. ubatubaensis*, share five synapomorphies: (1) body somites with dense setular surface ornamentation; (2) distal armature of antennary endopod composed of three geniculate and three non-geniculate elements; (3) P2–P4 coxae with outer spinous process; (4) P4 exopod one-segmented; and (5) female P5 exopod and baseoendopod fused into a single plate, with eight elements in total. *Laophontisochra terueae* expresses these characters except for character 3, i.e. the outer spinous process on the coxae of P2–P4 is absent in *L. terueae*. Based on the absence of this character alone, Björnberg (2014) assigned this species, not to *Acuticoxa*, but to *Laophontisochra*. This species also shares the biarticulated condition of the caudal seta V comprising swollen proximal part and setular distal part with all *Acuticoxa* species (cf. Huys and Kihara 2010), indicating a probable close affinity between these taxa. Thus, we propose to tentatively re-allocate *L. terueae* into *Acuticoxa* as *A. terueae* (Björnberg, 2014) comb. nov.

### Relationships amongst clade members with coxal outer projections on the thoracopods

The monophyly of the family Nannopodidae has been questioned by several researchers (e.g. Boxshall and Halsey 2004; Kihara and Huys 2009; Huys and Kihara 2010; Karanovic and Cho 2018). Por (1986) proposed the family Huntemanniidae and presented a brief diagnosis combining *Metahuntemannia*, *Huntemannia*, *Beckeria*, *Nannopus*, *Pontopolites* and *Pseudocletodes*, being unaware of the previous composition of Nannopodidae, which included *Nannopus*. Huys (2009) thereafter synonymised Huntemanniidae with Nannopodidae. Although the taxonomy, conceptualised by Por’s (1986), remains available for nannopodid copepods (Huys and Kihara 2010), this old familial diagnosis cannot satisfactorily accommodate the morphological range of nannopodid copepods because it is neither specific nor accurate. Since Por’s (1986) proposal, some nannopodid genera have been included and some excluded (see Dahms and Pottek 1992; Huys et al. 1996; Kihara and Huys 2009; Huys and Kihara 2010; Corgosinho 2012). Only three genera, *Huntemannia*, *Nannopus* and *Pontopolites* have remained in the family Nannopodidae, amongst which the genus *Pontopolites* remains questionable in that it differs from the familial diagnosis in having two-segmented antennary exopods and a natatorial P1 exopod. Based on its affinity with *Huntemannia*, which shares the presence of coxal processes on the P1, Huys and Kihara (2010) and Corgosinho (2012) included four genera, *Rosacletodes*, *Laophontisochra*, *Acuticoxa* and *Talpacoxa* in this family; however, both *Laophontisochra* and *Acuticoxa* show significant deviations from Por’s (1986) diagnosis, including female antennules with four segments, prehensile P1 and elongate caudal rami. These deviations imply that either the familial diagnosis should be extended or the phylogenetic relationship of the family members should be re-assessed.

Corgosinho (2012) suggested that *Huntemannia*, *Rosacletodes*, *Laophontisochra*, *Acuticoxa* and *Talpacoxa* form a clade within the family Nannopodidae; this relationship is supported by the presence of an outer coxal projection on the P1 and reduced P2–P4. However, there are morphological differences in the rostrum, male antennules, antennary endopod and exopod, both rami of the P1 and sexual dimorphism in the P3. It raises questions about the validity of this relationship amongst the five genera. George (2002) suggested that *L. maryamae* and *A. biarticulata* belong to a plesiomorphic lineage of the family Cristacoxidae. This argument was subsequently rejected by Huys and Kihara (2010) who provided contrary evidence showing a close relationship between *L. maryamae* and *A. biarticulata* and the nannopodid genus *Huntemannia*, rather than between those two species and any cristacoxid genera. These authors suggested that the presence of coxal projections on the P1–P4 is a significant synapomorphy between *Huntemannia* and *Laophontisochra*. However, a closer relationship between *Huntemannia* and *Nannopus* is evident. These two genera share the presence of anterior setules on the rostrum, the absence of geniculate distal elements on the antennary endopod, the uniramous mandibular palp, the non-prehensile P1 endopod, the short caudal rami and the shape of the sexually-dimorphic male P3. Karanovic and Cho (2018) noted that some species of both genera show different endopodal complements on the P3 between females and males, without any differences in segmentation. By contrast, the species of *Nannopus* display certain primitive characters, such as the presence of two abexopodal setae on the antennary allobasis (e.g. Fiers and Kotwicki 2013; Kim et al. 2017; Vakati and Lee 2017).

As reported by Huys and Kihara (2010), the prehensile P1 endopod is a significant synapomorphy for *Laophontisochra* and *Acuticoxa*. The new nannopodid genus, *Concilicoxa* gen. nov., described from the Yellow Sea of South Korea, also shares this character, as well as additional synapomorphies that provide evidence of affinity amongst these three genera: (1) the four-segmented female antennule and its elongate first segment (vs. five-segmented in other congeners); (2) antennary exopod and abexopodal seta rudimentary or missing (vs. a one-segmented exopod and presence of a developed abexopodal seta in other congeners); (3) the enlarged maxilliped with a geniculate claw-like endopod (vs. non-geniculate endopod in other congeners); (4) both coxae of the P2–P4 are connected by a large, broad intercoxal sclerite (vs. a small and narrow plate in other congeners); and (5) the female genital complex with a typical structure comprising a large copulatory pore and reverse ‘V’- or ‘U’-shaped genital slit (vs. small copulatory pore and transverse genital slit in other congeners).

Although the male of *Laophontisochra* remains unknown, a derived condition may be expressed in the male antennules–with a single compound segment distal to geniculation in *A. terueae* comb. nov. and *C. hispida* gen. et sp. nov. The male antennule of *T. brandini* possesses two segments distal to geniculation and the male antennule of *R. kuehnemanni* (Pallares, 1982) remains undescribed.

Huys and Kihara (2010) assumed that the P1 endopods in *Rosacletodes* and *Talpacoxa* are structurally identical with those of *Laophontisochra* and *Acuticoxa* in the segmentation and setal armature, even though there is a remarkable difference in the length of the first endopodal segment. However, a fundamental difference in exopods of *Rosacletodes* and *Talpacoxa* from those of *Laophontisochra* and *Acuticoxa*, as well as *Concilicoxa* gen. nov., is also readily recognised: the exopodal elements on the P1 are strong and enlarged in *Rosacletodes* and *Talpacoxa*, except for a single delicate one, but *Laophontisochra*, *Acuticoxa* and *Concilicoxa* gen. nov. have setiform or geniculate elements instead. Except for *A. ubatubaensis*, the setal pattern of the P1exp-2 is identical in *Laophontisochra*, *Acuticoxa* and *Concilicoxa* gen. nov., with one to three small outer setae, one stout and uniplumose outer seta, one delicate distal seta and one geniculate distal seta.

Given these characteristics, the clade with outer coxal projections on the P1–P4 can be subdivided into three groups: (1) *Huntemannia*, which is characterised by the presence of a setular group on the rostrum, a five-segmented female antennule, the absence of geniculate setae on the distal armature of the antennary endopod, a one-segmented antennary exopod with four setae, a one-segmented mandibular palp, non-prehensile P1 endopod, sexual dimorphism expressed in the number of elements on the distal segment of the male P3 endopod; (2) *Rosacletodes* and *Talpacoxa*, which are characterised by the absence of a setular group on the rostrum, a five-segmented female antennule, the presence of geniculate setae on the distal armature of the antennary endopod, a one-segmented antennary exopod with three setae, a two-segmented mandibular palp, prehensile short P1 endopod, P1 exopod with stout spines and presence of a sexually-dimorphic apophysis on the distal endopodal segment of the male P3; and (3) *Laophontisochra*, *Acuticoxa* and *Concilicoxa* gen. nov., which are characterised by the absence of a setular group on the rostrum, a four-segmented female antennule with elongation of the first segment, the presence of geniculate setae on the distal armature of the antennary endopod, the atrophied condition of the antennary exopod (represented by a single seta or absent), a two-segmented mandibular endopod, prehensile long P1 endopod, setiform elements on the P1 exopod and absence of sexual dimorphism in the male P3 (*A. terueae* comb. nov.) or development of a stout spine in the male P3 endopod (*C. hispida* gen. et sp. nov.) (Table 1).

**Table 1.** Comparison of morphological characters among nannopodid copepods with modified thoracopods for burrowing ability (female only).

						Setal armature										References
	A1	A2	Md		Mxl	P1		P2		P3		P4		P5		
	seg	expseg/setae	expseg/setae	enpseg/setae	expseg/setae	exp	enp	exp	enp	exp	enp	exp	enp	exp	enp	
*** Huntemannia ***																
*H. jadensis*	5	1/4	ab	fu/2	re/2	0.0.022	020	0.023	110	0.123	010	0.123	010	4–5	4	Sars 1909; Kornev and Chertoprud 2008
*H. micropus*	5	1/4	uk	uk	uk	0.023	020	0.021	010	0.021	010	0.022	010	4	4	Monard 1935
*H. lacustris*	uk	uk	uk	uk	uk	2–3 seg^1^	2^*^	0.5^*^	1(2)^*^	0.5(6)^*^	1(2)^*^	0.6^*^	1^*^	5	4	Wilson 1958
*H. biarticulatus*	5	1/4	uk	uk	uk	0.0.023	0.020	0.022	010	0.222	010	0.222	010	5	4	Shen and Tai 1973
*H. doheoni*	5	1/4	ab	fu/2	re/1	0.0.022	020	020^2^	010	011^2^	010	011^2^	010	5	4	Song et al. 2007
*** Rosacletodes ***																
*R. kuehnemanni*	6	1/3	fu/1	1/3	1/2	0.021	020	0.022	010	121	010	121	010	5	6	Pallares 1982
*** Laophontisochra ***																
*L. maryamae*	4	re/1	re/1	1/3	ab	0.023	0.011	0.022	ab	0.022	ab	0.022	010	4	1	George 2002
*** Acuticoxa ***																
*A. biarticulata*	4	1/1	uk	uk	uk	0.023	0.011	0.022	010	0.022	010	122	010	4	3	George 2002
*A. ubatubaensis*	4	ab	re/1	1/3	ab	0.023	0.011	023	010	023	010	023	010	4	2	Huys and Kihara 2010
*A. terueae* comb. nov.	4	ab ^3^	re/2	1/2^3^	ab	0.033^4^	0.020	0.011(2)	010	0.022	010	0.022^5^	010	4	3	Björnberg 2014
*** Talpacoxa ***																
*T. brandini*	5	1/3	1/1	1/3	1/2	023	0.020	0.020	010	0.121	010	0.121	010	5	3	Corgosinho 2012
***Concilicoxa* gen. nov.**																
*C. hispida* gen. et sp. nov.	4	re/1	ab	2/1.3	1/2	0.022	0.011	022	ab	112	000	112	010	4	1	the present study

Abbreviations: A1, antennule; A2, antenna; ab, absent; enp, endopod; exp, exopod; fu, fused to basis, Md, mandible; Mxl, maxillule; re, endopod or exopod represented by setae; seg, segmentation; uk, unknown. ^*^Total number of setae. ^1^The original description by Wilson (1958) provided only its segmentation. ^2^Exopods of the P2–P4 are one-segmented, but with a vestigial suture line between the two segments. ^3^Segmentation and armature of the male antenna and mandible were referred because those of females were damaged. ^4^Björnberg (2014) described the exopod bearing one seta on the first segment, and one lateral and three terminal setae on the second segment. However, the illustration of Björnberg (2014, fig. 11A) indicates an armature of 0.033. ^5^The male has a one-segmented exopod.

Corgosinho (2012) suggested that the development of the coxal outer process and the reduction of both rami in P2–P4, along with the strengthening of the outer exopodal elements in *Huntemannia*, *Rosacletodes*, *Laophontisochra*, *Acuticoxa* and *Talpacoxa* are the results of adaptation to a burrowing interstitial lifestyle. He also suggested that the burrowing ability of *Talpacoxa* was conferred by the remarkably-developed process of the P1 coxa and both compact and well-ornamented rami of the P1. It is likely that the specialised morphology of the intercoxal sclerite of P1, which is broad, elongate and bearing a transversal groove, can facilitate the burrowing activity (Corgosinho 2012: figs 4A, 6A). However, the morphology of the P2–P4 is relatively unsuitable for burrowing activity due to its weak outer elements and absence of the intercoxal sclerite. By contrast, three genera, *Laophontisochra*, *Acuticoxa* and *Concilicoxa* gen. nov., exhibit prehensile P1 endopods, with a small coxal projection, which is distinctly smaller than coxa, does not seem designed for burrowing. Instead, these three genera may have acquired a burrowing lifestyle by the development of stout and well-developed exopodal elements and large, broad intercoxal sclerites in P2–P4. Our comparison of the detailed morphology of thoracopods indicates that the P1 may play a role in the burrowing activity in *Talpacoxa*, whereas the P2–P4 confers this ability in *Laophontisochra*, *Acuticoxa* and *Concilicoxa* gen. nov. This hypothesis supports the subdivision of the nannopodid clade into three groups.

## Supplementary Material

XML Treatment for Concilicoxa

XML Treatment for Concilicoxa hispida
